# Simple and Rapid Colorimetric Detection of Canine Parainfluenza Virus 5 (*Orthorubulavirus mammalis*) Using a Reverse-Transcription Loop-Mediated Isothermal Amplification Assay

**DOI:** 10.3390/pathogens12070921

**Published:** 2023-07-08

**Authors:** Jong-Min Kim, Hye-Ryung Kim, Ji-Su Baek, Oh-Kyu Kwon, Hae-Eun Kang, Yeun-Kyung Shin, Choi-Kyu Park

**Affiliations:** 1 College of Veterinary Medicine & Animal Disease Intervention Center, Kyungpook National University, Daegu 41566, Republic of Korea; kjm51062@knu.ac.kr (J.-M.K.); gpfuddl25@knu.ac.kr (H.-R.K.); sy20103712@knu.ac.kr (J.-S.B.); 2 DIVA Bio Incorporation, Daegu 41519, Republic of Korea; 3 Foreign Animal Disease Division, Animal and Plant Quarantine Agency, Gimcheon 39660, Republic of Korea; kwonok59@korea.kr (O.-K.K.); kanghe@korea.kr (H.-E.K.)

**Keywords:** canine parainfluenza virus 5, reverse-transcription loop-mediated isothermal amplification, visual detection

## Abstract

Despite its many advantages, a reverse-transcription loop-mediated isothermal amplification (RT-LAMP) assay has yet to be developed for canine parainfluenza virus 5 (CPIV5). In this study, a visual RT-LAMP (vRT-LAMP) assay was developed for the rapid detection of CPIV5 in clinical samples. At a constant reaction temperature of 62 °C, the assay was completed within 40 min, and the results could be directly detected with the naked eye using a hydroxynaphthol blue (HNB) metal indicator without any additional detection apparatuses. The assay specifically amplified CPIV5 RNA with a limit of detection of 10 RNA copies/reaction, which was 10-fold more sensitive than the previously reported conventional reverse-transcription polymerase chain reaction (cRT-PCR) assay and was comparable to the previously reported real-time RT-PCR (qRT-PCR) assay. In a clinical evaluation using 267 nasopharyngeal swab samples collected from hospitalized dogs with respiratory symptoms, the CPIV5 detection rate using the vRT-LAMP assay was 5.24% (14/267), which was higher than that of the cRT-PCR assay (4.49%, 12/267) and consistent with that of the qRT-PCR assay, demonstrating 100% concordance with a kappa coefficient value (95% confidence interval) of 1 (1.00–1.00). The discrepancies in the results of the assays were confirmed to be attributed to the low sensitivity of the cRT-PCR assay. Owing to the advantages of a high specificity, rapidity, and simplicity, the developed vRT-LAMP assay using an HNB metal indicator will be a valuable diagnostic tool for the detection of CPIV5 in canine clinical samples, even in resource-limited laboratories.

## 1. Introduction

Parainfluenza virus 5 (PIV5), which is also known as *Orthorubulavirus mammalis*, is a nonsegmented, single-stranded, negative-sense RNA virus classified within the *Orthorubulavirus* genus in the *Rubulavirinae* subfamily of the *Paramyxoviridae* family. The viral genome comprises a 3′ leader region, a 5′ trailer region, and seven nonoverlapping genes, such as the nucleocapsid protein (N), V protein (V), phosphoprotein (P), matrix protein (M), fusion protein (F), small hydrophobic protein (SH), hemagglutinin-neuraminidase protein (HN), and large protein (L) or RNA polymerase [1].

Since its first isolation in 1956 from rhesus and cynomolgus monkey kidney cells [2], PIV5 has been identified in several host species, including humans, dogs, cats, pigs, calves, horses, and lesser pandas [3,4,5,6,7]. In dogs, canine PIV5 (CPIV5) was first identified in laboratory dogs demonstrating respiratory illness in 1967 [8]. Since then, CPIV5 has been globally distributed and is frequently detected as a primary pathogen associated with canine infectious respiratory disease (CIRD) [9,10,11,12]. In coyotes, ferrets, and rodents, the cross-species transmission of CPIV5 has been reported [13,14], and some studies have suggested that PIV5 may be a potential zoonotic pathogen based on the high genetic homology between human and animal PIV5 strains and the replicability of CPIV5 in human cell lines [3,15].

Given its global distribution and clinical effect on dogs as a primary pathogen in CIRD, CPIV5 has been routinely tested for etiological diagnoses in CIRD-affected dogs. Given the difficulty and time-consuming nature of virus isolation, the routine diagnosis of CPIV5 infections has relied on reverse-transcription polymerase chain reaction (RT-PCR)-based assays, such as conventional RT-PCR (cRT-PCR) [9,11,16], RT-nested PCR [17], and real-time RT-PCR (qRT-PCR) [10,12,18]. However, despite their high diagnostic sensitivity and specificity, these RT-PCR-based assays require specialized labor, complicated processes to detect amplified products, and sophisticated and expensive equipment, rendering such methods unsuitable for on-site diagnoses in field situations or resource-limited laboratories. Therefore, there is an urgent need for a simple, rapid, and cost-effective assay with a desirable diagnostic specificity and sensitivity for detecting CPIV5 in canine clinical samples.

Loop-mediated isothermal amplification (LAMP) technology has become a valuable molecular diagnostic tool since its first introduction in 2000 [19], with a high sensitivity, specificity, simplicity, and rapidity for the detection of various pathogens [20,21,22]. Moreover, the LAMP method can be employed as an on-site diagnostic assay and can be easily applied in local veterinary hospitals without specialized diagnostic facilities. LAMP can be performed using a simple constant-temperature water bath or heating block without a specialized thermocycler, and its results can be observed with the naked eye without any additional detection procedures [23,24,25]. Thus, several LAMP or reverse-transcription LAMP (RT-LAMP) assays have been developed for the diagnosis of infectious canine diseases, including canine distemper virus [26], canine parvovirus [27], *Babesia canis* [28], *Ehrlichia canis* [29], and *Hepatozoon canis* [30]. However, to the best of our knowledge, no RT-LAMP assay is currently available for CPIV5. Therefore, in this study, we first developed a visual RT-LAMP (vRT-LAMP) assay using a hydroxynaphthol blue (HNB) metal indicator for the rapid and simple detection of CPIV5 RNA and then evaluated the assay’s sensitivity, specificity, and applicability to canine clinical samples.

## 2. Materials and Methods

### 2.1. Samples and Nucleic Acid Extraction

A CPIV5 D008 vaccine strain with a viral titer of ≥10^4.0^ TCID_50_/mL was used to develop and optimize the vRT–LAMP conditions. Two other CPIV5 vaccine strains (Cornell and NL-CPI-5 strains), a Korean CPIV5 field isolate (KCPIV5-2301 strain) detected through a qRT-PCR assay [12], and seven other canine pathogens obtained from commercially available vaccines were used to evaluate the specificity of the developed assay. The canine pathogens were as follows: canine respiratory coronavirus (CRCoV, K37 strain), canine coronavirus (CCoV, K378 strain), canine influenza virus (CIV, A/Canine/Korea/01/07(H3N2)), canine distemper virus (CDV, Onderstepoort strain), canine adenovirus 2 (CAdV-2, Ditchfield strain), canine parvovirus (CPV, 7809 16-LP strain), and *Bordetella bronchiseptica* (*B. bronchiseptica*, S-55 strain). All pathogen samples were stored at −80 °C until use. For the clinical evaluation of the vRT-LAMP assay, a total of 267 nasopharyngeal swab samples were collected from dogs manifesting respiratory symptoms through a collaboration with a companion animal healthcare company (Postbio Co., Ltd., Guri, Republic of Korea) and a regional veterinary service laboratory (Daegu, Republic of Korea). A TANBead Nucleic Acid Extraction Kit for automated extraction (TANBead, Taoyuan, Taiwan) was used to immediately extract the total nucleic acids from the collected swab samples according to the manufacturer’s instructions. The extracted RNA or DNA samples were eluted in 100 μL of nuclease-free water, allocated to small volumes, and stored at −80 °C until use.

### 2.2. Construction of an RNA Standard

The CPIV5 N gene fragment was amplified by RT-PCR using an RNA template extracted from the D008 vaccine strain and a pair of primers (forward, 5′-CAGAGTAGTTCAATAAGGACCT-3′; reverse, 5′-CTAACCCGTCCGGGCCTA-3′), which were designed based on the sequence of the CPIV5 KCPIV5-2301 strain (GenBank accession No. OQ716700). The RT-PCR was performed using commercial RT (PrimeScript™ 1st strand cDNA Synthesis Kit, Takara Bio, Kusatsu, Shiga, Japan) and subsequent PCR (TaKaRa Ex Taq^®^, Takara Bio, Kusatsu, Shiga, Japan) kits according to the manufacturer’s instructions. The 1712 bp N gene fragment, containing all the cRT-PCR, qRT-PCR, and vRT-LAMP amplicons tested, was amplified, and subsequently purified using the Expin CleanUP SV kit (GeneAll Biotechnology, Seoul, Republic of Korea). The purified fragments were then cloned into the pTOP TA V2 vector using the TOPclone TA Core Kit (Enzynomics, Daejeon, Republic of Korea) by following the manufacturer’s instructions. The recombinant plasmid DNA samples were linearized through digestion with EcoRI (Takara Bio, Kusatsu, Shiga, Japan) and were then purified. The in vitro transcription of the purified samples was conducted using the RiboMAX Large-Scale RNA Production System-T7 (Promega, Fitchburg, WI, USA) by following the manufacturer’s instructions. The RNA concentrations were determined by measuring the absorbance at 260 nm using a NanoDrop Lite Spectrophotometer (Thermo Fisher Scientific, Waltham, MA, USA). The copy numbers of the RNA transcript were quantified using previously described methods [23]. Subsequently, 10-fold-diluted RNA samples (10^6^ to 10^0^ copies/reaction) were stored at −80 °C and used as CPIV5 RNA standards for determining the limit of detection (LOD).

### 2.3. Primers for vRT-LAMP Assay

**Figure 1:**
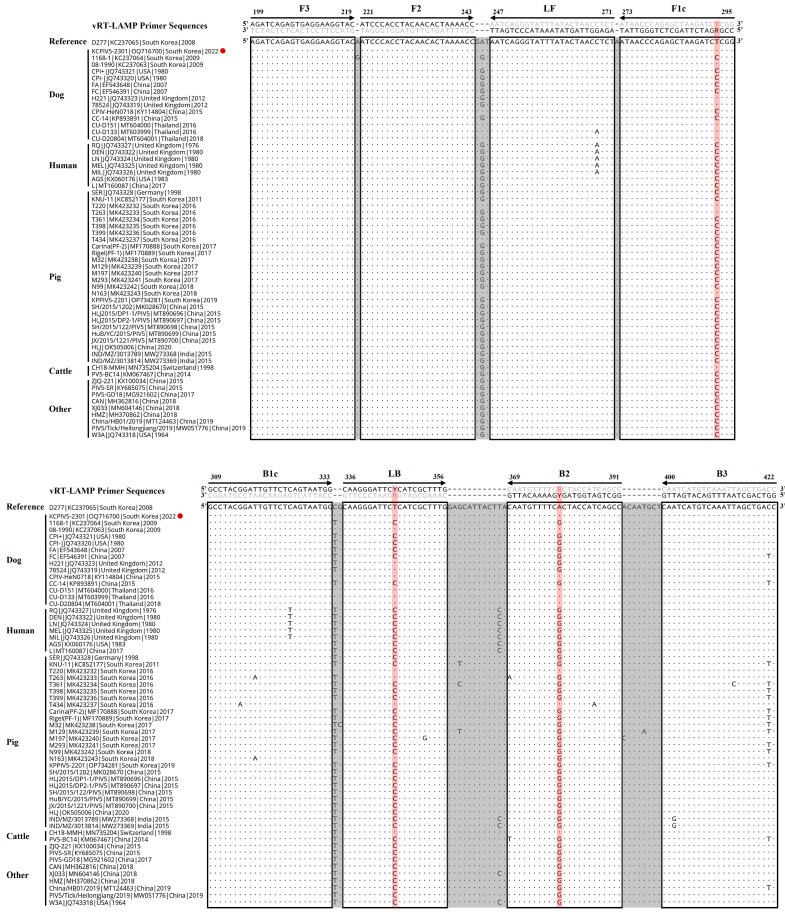
Alignment of the nucleocapsid protein (N) gene sequences of parainfluenza virus 5 (PIV5) strains derived from different hosts with the primer-binding sites of visual reverse-transcription loop-mediated isothermal amplification (vRT-LAMP). vRT-LAMP primer sequences are indicated along with the alignment and primer-binding sites are enclosed by boxes. A dot and letter indicate the same base and a variable base compared to the reference sequence (canine PIV5 D277 strain, GenBank accession No. KC237065), respectively. A red circle indicates the Korean CPIV5 field isolate (KCPIV5-2301 strain, GenBank accession No. OQ716700) used to evaluate the specificity of the developed vRT-LAMP assay. A red background line indicates the variable nucleotide sites where degenerate nucleotides (R or Y) were employed on the F1c, LB, or B2 primer to match the corresponding viral target gene sequence.

To detect a broad spectrum of PIV5 strains from different hosts [3,31] using the vRT-LAMP assay, the N gene sequences of 59 PIV5 strains (15 canine, 7 human, 26 swine, and 11 other host strains) were retrieved from the GenBank database of the National Center for Biotechnology Information (NCBI). Conserved nucleotide sequences within the N gene were identified by conducting multiple alignments using the BioEdit Sequence Alignment Editor program (https://www.mbio.ncsu.edu/BioEdit/bioedit.html, accessed on 7 March 2023). With these conserved sequences, a set of six primers for the vRT-LAMP assay, including two outer primers (F3 and B3), two inner primers (FIP and BIP), and two loop primers (LF and LB), was manually designed using Primer Explorer V5 software (Fujitsu System Solutions Ltd., Tokyo, Japan). For the variable nucleotide sites on the sequences of the F1c, LB, and B2 primers, each nucleotide was changed to a degenerated nucleotide (Y or R) to match the corresponding viral target gene sequence. Using NCBI BLAST (https://blast.ncbi.nlm.nih.gov/Blast.cgi, accessed on 15 March 2023), the specificity of the primers for vRT-LAMP was evaluated through sequence similarity searches and the primer sequences of the primers were confirmed to almost match the N gene sequences of different PIV5 strains (Figure 1). All primers were synthesized by a commercial company (BIONICS, Seoul, Republic of Korea) (Table 1).

### 2.4. Optimization of vRT-LAMP Conditions

The vRT-LAMP conditions were optimized with a commercial vRT-LAMP kit (Mmiso^®^ RNA Amplification Detection Kit, M monitor, Daegu, Republic of Korea) that contained HNB in the reaction buffer to visually detect the RT-LAMP results. HNB is a metal ion indicator that imparts a purple color to the LAMP reaction solution containing Mg^2+^ ions and dNTPs before the reaction starts. During amplification, insoluble magnesium pyrophosphate is formed, causing a decrease in the Mg^2+^ ion concentration in the solution. Thereafter, the reaction solution color changes from purple (negative reaction) to sky blue (positive reaction) [32]. The 25 μL reaction mixture, containing 12.5 μL of 2× reaction buffer, 1 μL of enzyme mix, 1.6 μM of the inner primers (FIP and BIP), 0.2 μM of the outer primers (F3 and B3), 0.8 μM of the loop primers (LF and LB), 5 μL of the RNA template, and dH_2_O added to reach the final volume, was prepared according to the manufacturer’s instructions. As previously described [23,24,25], the vRT-LAMP conditions were optimized by performing the amplification at different reaction temperatures (54 °C–68 °C) and different reaction times (30–60 min). All experiments were performed in triplicate. By observing the color change of the reaction from purple to sky blue owing to the presence of HNB, the assay results were visually detected [32]. Furthermore, the amplified products were subjected to 1.5% agarose gel electrophoresis, stained with NEO green dye (Neoscience, Suwon, Republic of Korea), and detected by observing LAMP-specific ladder-like patterns of DNA bands using an ultraviolet light transilluminator (Bio-Rad, Hercules, CA, USA).

### 2.5. Reference cRT-PCR and qRT-PCR Assays

To compare the diagnostic performance of vRT-LAMP, previously described cRT-PCR [11] and qRT-PCR [10] assays targeting the same CPIV5 N gene were adopted as reference assays. The cRT-PCR assay was conducted using a commercial RT-PCR kit (One-Step RT-PCR Premix, Inclone Biotech, Yongin, Republic of Korea) with N gene-specific primers (forward, 5′- AGTTTGGGCAATTTTTCGTCC -3′; reverse, 5′- TGCAGGAGATATCTCGGGTTG -3′) in a 50 μL reaction mixture containing 25 μL of 2X premix, 2 μL of enzyme mix, 0.4 μM of each primer, 5 μL of the RNA template, and dH_2_O to reach the final volume, according to the manufacturer’s instructions. Amplification was conducted using a thermal cycler (Applied Biosystems, Foster City, CA, USA), and the PCR protocol was as follows: reverse transcription at 50 °C for 50 min, initial denaturation at 95 °C for 15 min followed by 40 cycles of amplification (95 °C for 20 s, 55 °C for 40 s, and 72 °C for 45 s), and a final extension at 72 °C for 5 min. The expected 667 bp amplicons were visualized using 1.5% agarose gel electrophoresis and staining with a NEO green dye (Neoscience, Suwon, Republic of Korea). qRT-PCR was performed using a commercial one-step real-time RT-PCR kit (THUNDERBIRD™ Probe One-step qRT-PCR kit, Toyobo, Osaka, Japan) with N gene-specific primers (forward, 5′- GATCATTCCGCTTAATCCCC -3′; reverse, 5′- TTCTGCAAGTGCAGCATAGG -3′) and a TaqMan probe (5′-FAM-TCGTTCAGGTATGAGCCGTGGA-BHQ1-3′) in a 20 μL reaction mixture containing 10 μL of 2X reaction buffer, 0.5 μL of RT enzyme mix, 0.5 μL of DNA polymerase, 0.5 μM of each primer, 0.15 μM of probe, 5 μL of the RNA template, and dH_2_O to reach the final volume, according to the manufacturer’s instructions. A CFX96 Touch™ Real-Time PCR Detection System (Bio-Rad, Hercules, CA, USA) was used to perform the reaction, and the PCR protocol was as follows: reverse transcription at 50 °C for 10 min, initial denaturation at 95 °C for 1 min, and 40 cycles of amplification (denaturation at 95 °C for 15 s and annealing and extension at 60 °C for 45 s). At the end of each annealing step, the real-time fluorescence values for the FAM-labeled probes were measured in ongoing reactions. To interpret the qRT-PCR results, samples producing a cycle threshold (Ct) of ≤40 were considered positive, whereas those without Ct values were considered negative.

### 2.6. Specificity and Sensitivity of the vRT-LAMP Assay

To evaluate its specificity, the vRT-LAMP assay was performed using nucleic acids extracted from the stocks of the CPIV5 KCPIV5-2301 strain, three CPIV5 vaccine strains (D008, Cornell, and NL-CPI-5), seven other canine pathogens (CRCoV, CCoV, CIV, CDV, CAdV-2, CPV, and *B. bronchiseptica*), a canine-origin cell culture (MDCK cell), and a negative control (nuclease-free water). Using 10-fold serial dilutions (10^6^–10^0^ copies/reaction) of the standard RNA samples of the CPIV5 N gene, the LOD of the vRT-LAMP assay for CPIV5 was determined. Subsequently, the LOD of the vRT-LAMP assay was compared with those of the cRT-PCR [11] and qRT-PCR [10] assays using the RNA templates described above.

### 2.7. Comparative Evaluation of the vRT-LAMP Assay with Clinical Samples

A total of 267 nasopharyngeal swab samples were examined using the newly developed vRT-LAMP assay to clinically evaluate the vRT-LAMP assay, and the results were compared with those of the cRT-PCR and qRT-PCR assays [10,11]. The concordance between the vRT-LAMP and cRT-PCR or qRT-PCR results was analyzed using Cohen’s kappa statistics with a 95% confidence interval (CI). When the calculated kappa coefficient value (κ) was 0.81–1.0, the results were interpreted as an almost perfect agreement [33].

## 3. Results

### 3.1. Optimized Conditions of the vRT-LAMP Assay

**Figure 2:**
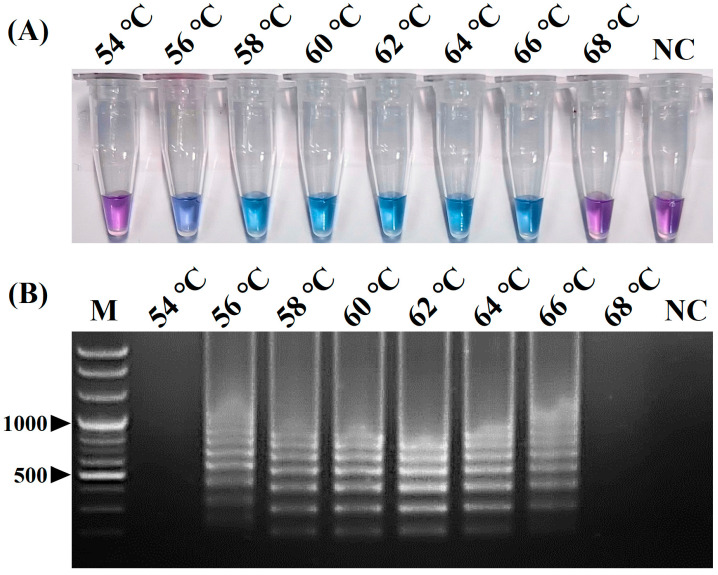
Optimal reaction temperature of the visual reverse-transcription loop-mediated isothermal amplification (vRT-LAMP) assay for detecting canine parainfluenza virus 5. (**A**) Colorimetric detection of the vRT-LAMP results. A transition in the color of the reaction solution, shifting from purple (negative reaction) to sky blue (positive reaction), indicates a positive reaction. (**B**) Electrophoretic analysis of the vRT-LAMP amplification products. Lane M, 100 bp plus DNA ladder. In the vRT-LAMP-positive reactions, LAMP-specific ladder-like electrophoresis patterns were observed. NC, negative control (nuclease-free water).

**Figure 3:**
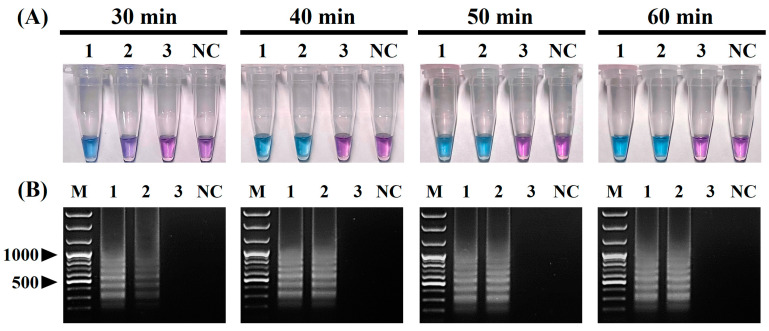
Optimal reaction time of the visual reverse-transcription loop-mediated isothermal amplification (vRT-LAMP) assay for detecting canine parainfluenza virus 5. (**A**) Colorimetric detection of the vRT-LAMP results. A transition in the color of the reaction solution, shifting from purple (negative reaction) to sky blue (positive reaction), indicates a positive reaction. (**B**) Electrophoretic analysis of the vRT-LAMP-amplified products. Lane M, 100 bp plus DNA ladder. In the vRT-LAMP-positive reactions, LAMP-specific ladder-like electrophoresis patterns were observed. Tubes/lanes 1–3, vRT-LAMP results for CPIV5 N gene RNAs (from 10^2^ to 10^0^ copies/reaction) at four reaction times: 30, 40, 50, and 60 min. NC, negative control (nuclease-free water).

The positive color change from purple (negative reaction) to sky blue (positive reaction) of the reaction solution and the intensity of the positive sky-blue color and ladder-like DNA bands generated through the electrophoresis of the vRT-LAMP products were observed for judging the optimized reaction conditions for the vRT-LAMP assay. After the vRT-LAMP reaction with the CPIV5 N gene RNA templates (10^3^ copies/reaction), a positive color change from purple to sky blue in the reaction tubes was observed within the temperature range from 56 °C to 66 °C (Figure 2A), and a ladder-like pattern of DNA bands was generated via gel electrophoresis (Figure 2B), indicating the successful production of stem-loop DNA with the inverted repeats of the target sequence. However, at a reaction temperature of 62 °C, the clearest color changes and electrophoretic bands were observed. Subsequently, the vRT-LAMP assay was conducted with three dilutions of the CPIV5 N gene RNA samples with different copy numbers (10^2^, 10^1^, and 10^0^ copies/reaction) for different reaction times ranging from 30 to 60 min at 62 °C. Within 30 min, positive results were observed; however, the optimal reaction time, defined as the minimum time required to reach the LOD based on the clear and distinct amplicon bands observed in the reaction using 10^1^ copies/reaction of the CPIV5 N gene RNA template, was determined to be 40 min (Figure 3A,B). These results determined the optimal reaction temperature and time for the CPIV5 vRT-LAMP assay as 62 °C and 40 min, respectively, and subsequent experiments were performed using these optimized reaction conditions.

### 3.2. Specificity of the vRT-LAMP Assay

**Figure 4:**
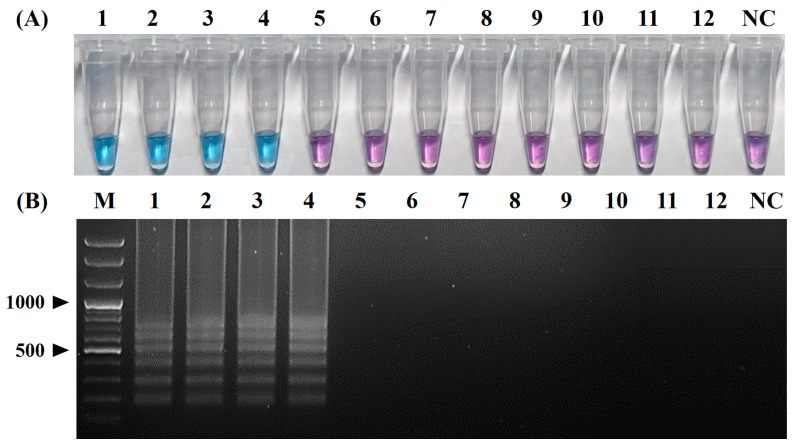
Specificity of the visual reverse-transcription loop-mediated isothermal amplification (vRT-LAMP) assay for canine parainfluenza virus 5. (**A**) Colorimetric detection of the vRT-LAMP results. A transition in the color of the reaction solution, shifting from purple (negative reaction) to sky blue (positive reaction), indicates a positive reaction. (**B**) Electrophoretic analysis of vRT-LAMP-amplified products. Lane M, 100 bp plus DNA ladder. LAMP-specific ladder-like electrophoresis patterns were observed in vRT-LAMP-positive reactions. Tubes and lanes 1–4: canine parainfluenza virus 5 (CPIV5) Korean field strain KCPIV5-2301 and CPIV5 vaccine strains D008, Cornell, and NL-CPI-5, respectively. Tubes and lanes 5–12: canine respiratory coronavirus, canine coronavirus, canine influenza virus, canine distemper virus, canine adenovirus 2, canine parvovirus, *Bordetella bronchiseptica*, and MDCK cells, respectively. Tube and lane NC, negative control (nuclease-free water).

Positive colorimetric reactions (from purple to sky blue) were observed only in the reaction tubes containing the RNA templates of four CPIV5 strains (tube Nos. 1, 2, 3, and 4), whereas the reaction tubes containing the nucleic acid templates of the other seven canine pathogens (CRCoV, CCoV, CIV, CDV, CAdV-2, CPV, and *B. bronchiseptica*) and a canine-origin cell culture (MDCK cells) remained purple (Figure 4A). Typical ladder-like band patterns were also obtained via an electrophoretic analysis only in reactions with the RNA templates of the four CPIV5 strains, whereas no amplicon was observed in reactions with the nucleic acid templates of the seven other canine pathogens and NC (Figure 4B). These results indicate that the primer set used in the vRT-LAMP assay was highly specific for the amplification of the CPIV5 N gene.

### 3.3. Sensitivities of the vRT-LAMP, cRT-PCR, and qRT-PCR Assays

**Figure 5:**
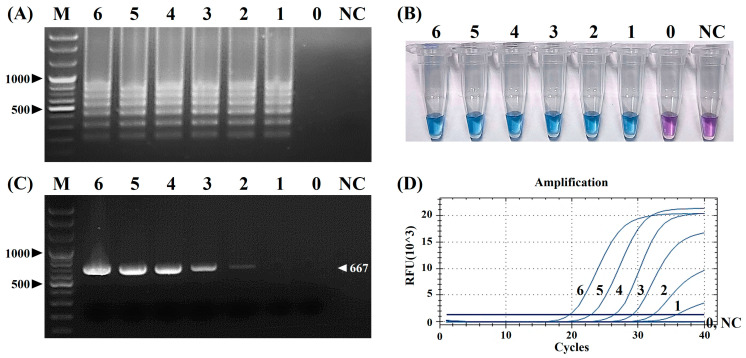
Comparison of the analytical sensitivities of the visual reverse-transcription loop-mediated isothermal amplification (vRT-LAMP), conventional reverse-transcription polymerase chain reaction (cRT-PCR), and real-time RT-PCR (qRT-PCR) assays to canine parainfluenza virus 5. (**A**,**B**) Electrophoretic analysis and visualization of the vRT-LAMP results. (**C**) Electrophoretic analysis of the cRT-PCR assay. (**D**) Amplification curves of the qRT-PCR assay. The numbers in (**A**–**D**) represent serial 10-fold dilutions of the standard canine parainfluenza virus 5 N gene RNAs (from 10^6^ to 10^0^ copies/reaction, lines/tubes 6–0); lane M, 100 bp plus DNA marker; tube and lane NC, negative control (nuclease-free water).

The LOD of the vRT-LAMP assay was evaluated using serial dilutions of the CPIV5 N gene RNA standards and compared with those of the cRT-PCR and qRT-PCR assays. The LODs of the vRT-LAMP, cRT-PCR, and qRT-PCR assays were then determined as 10^1^ (Figure 5A,B), 10^2^ (Figure 5C), and 10^1^ copies/reaction (Figure 5D), respectively. Regarding sensitivity, the vRT-LAMP assay was 10-fold more sensitive than the cRT-PCR assay and was comparable to the qRT-PCR assay. These results reveal that the vRT-LAMP assay is highly sensitive and is suitable for CPIV5 detection (Figure 5).

### 3.4. Diagnostic Performance of the vRT-LAMP Assay for Clinical Samples

Of the 267 nasopharyngeal swab samples, 14, 12, and 14 samples were confirmed as CPIV5 RNA-positive by the vRT-LAMP, cRT-PCR, and qRT-PCR assays, respectively (Table 2). The CPIV5 detection rates of the vRT-LAMP and qRT-PCR assays were the same at 5.24%, which was higher than that of the cRT-PCR assay at 4.49%. The rates of the positive, negative, and overall agreements between the results of the vRT-LAMP and qRT-PCR assays were 100% (14/14), 100% (253/253), and 100% (267/267), respectively, with a κ value of 1.00 (1.00–1.00), indicating that the results of the two assays had perfect agreement. In comparison, all 12 CPIV5-positive samples detected by the cRT-PCR assay were also CPIV5-positive as detected by the vRT-LAMP and qRT-PCR assays, and the vRT-LAMP and qRT-PCR assays further detected CPIV5 from two cRT-PCR-negative samples, indicating that the vRT-LAMP and qRT-PCR assays had a better clinical diagnostic sensitivity than the cRT-PCR assay. For the two discordant clinical samples that were vRT-LAMP-positive and cRT-PCR-negative, the DNA sequences of the vRT-LAMP amplicons were analyzed using the F3 and B3 primers of vRT-LAMP with Sanger sequencing by a commercial company (BIONICS, Seoul, Republic of Korea). All nucleotide sequences of the 224 bp fragment were identical to the corresponding sequences of the CPIV5 N gene of the CPIV5 KCPIV5-2301 strain. On determining the Ct values of the 14 CPIV5-positive samples according to the vRT-LAMP and qRT-PCR assays, the 12 concordant (cRT-PCR-positive) samples had Ct values of ≤32.43 (equivalent to 153 RNA copies of the target genes) and the two discordant (cRT-PCR-negative) samples had Ct values of ≥34.52 (equivalent to 34 RNA copies of the target genes) (Table 2).

## 4. Discussion

Nowadays, several LAMP assays have been developed and widely used to detect various animal and human pathogens given their high specificity, sensitivity, rapidity, and simplicity, which satisfy the World Health Organization (WHO)’s criteria for diagnostic tests [20,22]. Several LAMP or RT-LAMP assays have been developed to detect canine pathogens [26,27,28,29,30]; however, no RT-LAMP assays have been developed to detect CPIV5. Therefore, in this study, we first developed a vRT-LAMP assay that can visually detect CPIV5 RNA and then comparatively evaluated the diagnostic performance of the assay with previously described cRT-PCR and qRT-PCR assays using canine clinical samples.

The vRT-LAMP assay developed yields several advantages that fulfill the WHO’s ASSURED (Affordable, Sensitive, Specific, User-friendly, Rapid and robust, Equipment-free, and Deliverable to end-users) criteria for an ideal molecular diagnostic test for field application [34]. First, all the steps of the developed vRT-LAMP assay can be conducted at a constant reaction temperature due to the use of the strand-displacing *Bst* DNA polymerase, which allows the operation of the assay via simple instruments such as a water bath or dry heat block [20,21]. Moreover, the amplified results of the assay can be directly monitored with the naked eye by observing the color change of the reaction tube, which is attributed to the HNB metal indicator in the reaction mix [32]. These simple amplification and monitoring steps render the vRT-LAMP assay affordable and easily applicable in the field without any expensive thermocycling or monitoring instruments.

The original LAMP method, first introduced by Notomi et al. [19], was operated using four basic LAMP primers, including two outer primers (F3 and B3) and two inner primers (FIP and BIP). Subsequently, Nagamine et al. [35] developed a more advanced LAMP method using additional loop primers (LF and LB) that can accelerate the LAMP reaction, improving the efficiency and sensitivity of the LAMP method. Since then, most LAMP methods have been developed using six primers, including loop primers, to achieve a higher sensitivity and faster turnaround times [23,24,25]. In this study, we also tried to design six primers for the development of vRT-LAMP assays for CPIV5 based on the highly conserved CPIV N gene sequences and successfully designed three pairs of vRT-LAMP primers, including two pairs of basic LAMP primers (F3 and B3, and FIP and BIP) and a pair of loop primers (LF and LB), which accelerated the vRT-LAMP reaction [35] (Figure 1 and Table 1). These primers recognize eight unique sites on the target CPIV5 N gene sequences, allowing the assay to be highly specific for the detection of CPIV5 (Figure 4). Under optimized reaction conditions, the vRT-LAMP was completed in 40 min and the assay results were directly detected with the naked eye using HNB as a colorimetric indicator, without any additional detection processes (Figure 2 and Figure 3), which was much faster than the qRT-PCR and cRT-PCR assays, which required at least 2 or 3 h of turnaround time [10,11]. The analytical sensitivity of the vRT-LAMP assay was higher than that of the previously used cRT-PCR assay [11] and was comparable to that of the previously well-established qRT-PCR assay [10]. In this study, the use of well-designed primers, including loop primers, resulted in a high specificity, sensitivity, and rapidity of the developed vRT-LAMP assay.

However, despite all the advantages of the developed CPIV5 vRT-LAMP assay, some bottlenecks remain to be overcome to fully satisfy the WHO’s ASSURED criteria. First, all reagents required for the vRT-LAMP assay require storage at −20 °C, and such a cold chain does not satisfy the criterion “Deliverable to end-users”. Recently, a dry LAMP system that uses dried or lyophilized reagents has been established for the on-site diagnosis of human and animal pathogens, averting the need for a cold chain and meeting the criterion of “Deliverable to end-users” [36,37,38,39]. Second, one of the most insurmountable challenges in establishing an on-site molecular diagnostic assay is the nucleic acid extraction and purification process. However, compared to the PCR-based assays, LAMP assays are inherently superior in overcoming this limitation, since LAMP using *Bst* DNA polymerase is less sensitive to inhibitors in a clinical sample solution than PCR using *Taq* DNA polymerase [40,41]. Thus, recent reports highlighting that direct LAMP assays can be performed using crude samples without complicated nucleic acid extraction and purification processes are noteworthy [42,43,44]. Therefore, further studies are warranted to convert this assay into a dry LAMP system combined with a simple nucleic acid extraction method to expand the on-site applicability.

The usefulness of the vRT-LAMP assay was further evaluated using 267 nasopharyngeal swab samples collected from hospitalized dogs exhibiting clinical signs of CIRD. The detection rates of CPIV5 for the vRT-LAMP assay and previous qRT-PCR assay were the same at 5.24% (14/267), showing 100% concordance between both assays with a kappa value (95% CI) of 1.0 (Table 2). On the contrary, the previous cRT-PCR assay failed to detect two clinical samples that were positive according to both the vRT-LAMP and qRT-PCR assays, resulting in a detection rate of 4.49% (12/267) (Table 2). The discrepancies between the assays occurred for two clinical samples; the Ct values of the 14 CPIV5-positive clinical samples were confirmed, and the Ct values of the 12 concordant samples (CPIV5-positive according to all three assays) were compared with those of the two discordant samples (CPIV5-positive according to both vRT-LAMP and qRT-PCR, but CPIV5-negative according to cRT-PCR). The Ct values of the 12 concordant samples were all ≤32.43 (equivalent to 153 RNA copies of the target genes); however, the Ct values of the two discordant clinical samples were all ≥34.52 (equivalent to 34 RNA copies of the target genes) (Table 2). Thus, the cRT-PCR assay failed to detect the two discordant clinical samples because of its low sensitivity, which is consistent with the LOD of the cRT-PCR assays, determined as 10^2^ RNA copies/reaction in the sensitivity evaluation (Figure 5). These clinical evaluation results show that the vRT-LAMP assay was highly specific and sensitive for CPIV5 detection in clinical samples, serving as an alternative diagnostic tool for the diagnosis of CPIV5 that can replace the previously described RT-PCR-based assays.

Given the broad host range and the possibility of cross-species transmission of the virus [3,13,14,15], a new molecular diagnostic assay for CPIV5 that can detect all PIV5 strains derived from different susceptible hosts is desirable. To address this issue, based on all 59 available PIV5 sequences, the primers for the vRT-LAMP assay were carefully designed for detecting all PIV5 strains from dogs and other host species (Figure 1). However, the diagnostic performance of the vRT-LAMP assay was evaluated only with canine clinical samples. Therefore, further clinical evaluation studies using non-canine clinical samples are necessary to fully utilize the vRT-LAMP assay in the field.

In summary, we first developed a vRT-LAMP assay using an HNB metal indicator that can visually detect CPIV5 RNA. The vRT-LAMP reaction was rapidly completed within 40 min at a constant reaction temperature of 62 °C without the use of a complicated and expensive thermocycler, which allows the vRT-LAMP assay to be conducted using a simple and cheap apparatus such as a water bath or heating block. Furthermore, the vRT-LAMP assay results can be easily judged with the naked eye after the reaction without any detection apparatuses or processes. These characteristics of the vRT-LAMP assay are in line with the WHO’s criteria for ideal diagnostic tests that can be used even in resource-limited laboratories. Given the high specificity, rapidity, and simplicity of the vRT-LAMP assay, it will be a valuable diagnostic tool for CPIV5 detection in canine clinical samples even in resource-limited laboratories.

## Figures and Tables

**Table 1:** vRT-LAMP primers used to amplify the N gene of canine parainfluenza virus 5.

Primer Name	Sequence (5′–3′) ^a^	Length (bp)	Genome Position ^b^
CPIV5N-F3	AGATCAGAGTGAGGAAGGTAC	21	119–219
CPIV5N-B3	GGTCAGCTAATTTGACATGATTG	23	400–422
CPIV5N-LF	AGAGGTTAGTATAAATACCCTGATT	25	247–271
CPIV5N–LB	CAAGGGATTC**Y**CATCGCTTTG	21	336–356
CPIV5N–FIP (F1c + F2)	CCG**R**GATCTTAGCTCTGGGTTAT + ATCCCACCTACAACACTAAAACC	46	273–295 + 221–243
CPIV5N–BIP (B1c + B2)	GCCTACGGATTGTTCTCAGTAATGG + GGCTGATGGTAG**Y**GAAAACATTG	48	309−333 + 369−391

^a^ Bold text in CPIV5N–LB, CPIV5N–FIP (F1c), and CPIV5N–BIP (B2) sequences represents a degenerate base: Y for C or T and R for A or G. ^b^ Locations of all primer sequences for the visual reverse-transcription loop-mediated isothermal amplification (vRT-LAMP) assay were derived from the complete genome sequence of the canine parainfluenza virus 5 (CPIV5) D277 strain (GenBank accession No. KC237065).

**Table 2:** Comparative diagnostic results for the detection of canine parainfluenza virus 5 in clinical samples.

Method		New vRT-LAMP			Positive Rate	Overall Agreement ^a^
		Positive	Negative	Total		
cRT-PCR [11]	Positive	12	0	12	4.49%	99.3%
	Negative	2	253	255		
	Total	14	253	267		
	Positive rate	5.24%				
qRT-PCR [10]	Positive	14	0	14	5.24%	100%
	Negative	0	253	253		
	Total	14	253	267		
	Positive rate	5.24%				

cRT-PCR, conventional reverse-transcription polymerase chain reaction; qRT-PCR, real-time RT-PCR; vRT-LAMP, visual reverse-transcription loop-mediated isothermal amplification. ^a^ The rates of positive, negative, and overall agreements for the vRT-LAMP assay relative to the qRT-PCR and cRT-PCR assays were 100% (14/14), 100% (253/253), and 100% (267/267), and 85.7% (12/14), 100% (253/253), and 99.3% (265/267), respectively. On determining the Ct values of the 14 canine parainfluenza virus 5 (CPIV5)-positive samples, the 12 concordant samples (CPIV5-positive according to all three assays) had Ct values of ≤32.43 (equivalent to 153 RNA copies of target genes), and the 2 discordant samples (CPIV5-positive according to vRT-LAMP and qRT-PCR, but CPIV5-negative according to cRT-PCR) had Ct values of ≥34.52 (equivalent to 34 RNA copies of the target genes).

## Data Availability

Not applicable.
